# Vitamin D Receptor Deficiency Does Not Affect Blood Pressure and Heart Function

**DOI:** 10.3389/fphys.2019.01118

**Published:** 2019-08-29

**Authors:** Sarah M. Grundmann, Alexandra Schutkowski, Barbara Schreier, Sindy Rabe, Bettina König, Michael Gekle, Gabriele I. Stangl

**Affiliations:** ^1^Institute of Agricultural and Nutritional Sciences, Martin Luther University Halle-Wittenberg, Halle (Saale), Germany; ^2^Competence Cluster for Nutrition and Cardiovascular Health (nutriCARD), Halle-Jena-Leipzig, Leipzig, Germany; ^3^Julius Bernstein Institute of Physiology, Faculty of Medicine, Martin Luther University Halle-Wittenberg, Halle (Saale), Germany

**Keywords:** vitamin D, vitamin D receptor, blood pressure, heart function, mice

## Abstract

Vitamin D is thought to play a role in blood pressure regulation, which in turn can influence cardiovascular risk. Several meta-analyses of cohort studies found low serum levels of 25-hydroxyvitamin D to be associated with increased blood pressure or increased cardiovascular morbidity and mortality in the general population. Active vitamin D mediates its function via the vitamin D receptor (Vdr), which is a ligand-activated transcription factor. A suitable model to examine the causal role of vitamin D in blood pressure regulation and heart function is the Vdr knockout (Vdr^–/–^) mouse. To elucidate the role of vitamin D on blood pressure, heart function, and cardiac myocyte size, we conducted a long-term study using Vdr^–/–^ mice and well-defined diets. Group 1 comprised Vdr^–/–^ mice that received a high-calcium, high-phosphorus rescue diet to prevent hypocalcemia and a rickets phenotype. Groups 2 and 3 included Vdr^+/+^ mice that were fed either the rescue diet or a control diet containing normal amounts of these minerals. As Vdr is a nuclear factor that regulates transcription, we analyzed the renal mRNA expression and serum concentration of renin and found that the Vdr^–/–^ group had an almost 50% higher renin mRNA expression in the kidney compared to both groups of Vdr^+/+^ mice. Additionally, serum concentration of renin in Vdr^–/–^ mice was significantly higher than that of Vdr^+/+^ mice that received the rescue or control diet (+ 17%,+ 32%; *P* < 0.05). In contrast, renin activity was lower in Vdr^–/–^ mice than in both groups of Vdr^+/+^ mice (*P* < 0.05). However, blood pressure, heart rate, cardiac myocyte sizes, and the expression of renal renin receptor, hepatic angiotensinogen and angiotensin II receptor, type 1, in kidney, liver and heart, did not differ between the three groups of mice. Additionally, data from transthoracic echocardiography did not indicate the role of Vdr on heart function, as the left ventricular ejection fraction, fractional shortening, and velocity of blood flow were comparable between the three groups. To conclude, the roles of Vdr and therefore most probably of vitamin D, in blood pressure regulation and heart function, were not confirmed by our findings.

## Introduction

Cardiovascular diseases (CVD) are the main cause of death worldwide (Roth et al., 2017). Several risk factors, including hypertension, can cause an early onset and accelerated progression of CVD. Besides pharmaceutical interventions, multiple dietary factors can affect blood pressure and, in turn, CVD risk. Dietary changes that have been shown to be successful in lowering blood pressure are weight loss, reduced sodium intake, increased potassium intake, vegetarian diet patterns, and dietary concepts such as the Dietary Approaches to Stop Hypertension-style (Appel, 2017). Another factor which has been linked to blood pressure and CVD is vitamin D (Artaza et al., 2009).

Most data on vitamin D and CVD risk come from studies involving patients who suffer from chronic kidney diseases. In such patients, the following two factors are relevant: (i) vitamin D-deficiency because the kidney is the major site for conversion of vitamin D to its active form (Nigwekar et al., 2012); and (ii) high risk for CVDs because the kidney is involved in blood pressure regulation (Gansevoort et al., 2013). Supplementation of patients suffering from chronic kidney diseases with vitamin D has been shown to reduce CVD mortality in these patients (Park et al., 1999; Li et al., 2015; Kumar et al., 2018). In addition to patients data, several meta-analyses of cohort studies found low serum levels of 25-hydroxyvitamin D [25(OH)D] to be associated with increased blood pressure (Scragg et al., 2007) or increased cardiovascular morbidity and mortality in the general population (Chowdhury et al., 2014; Schöttker et al., 2014; Gaksch et al., 2017).

In contrast to cohort studies, data obtained from intervention trials are less consistent. Some studies report beneficial effects of vitamin D supplementation on blood pressure in patients with a low vitamin D level (Bricio-Barrios et al., 2016), whereas most data obtained from intervention trials, in which individuals received a vitamin D supplement, show no effect of vitamin D on blood pressure (Pilz et al., 2015; McMullan et al., 2017; Tomson et al., 2017). This was also confirmed by a recent meta-analysis of randomized controlled human intervention trials that did not show a beneficial effect of vitamin D supplementation on blood pressure (Beveridge et al., 2018). The contradictory findings from association and intervention studies can, at least partly, be attributed to the following reasons. Sources of vitamin D in association and intervention studies differ. Intervention studies normally investigate the impact of orally administered vitamin D, while association studies focus on serum 25(OH)D, which comes mainly from the cutaneous synthesis of vitamin D via exposure to sunlight. Besides vitamin D synthesis, sunlight is capable of producing vasodilating nitric oxide (Weller, 2016) or can lower blood pressure via radiant heat (Sinha et al., 2010). Thus, it is possible that vitamin D-independent effects of sunlight are responsible for blood pressure reduction. Another key factor is that individuals with high physical outdoor activities have higher serum concentrations of 25(OH)D than less active individuals. As physical activity can reduce blood pressure and CVD risk irrespective of vitamin D (Bijnen et al., 1998; Brock et al., 2010), it is likely that the association between 25(OH)D and blood pressure is not causal.

More than 30 cell types and tissues have been reported to express the Vdr, suggesting that vitamin D action is not limited to the regulation of mineral homeostasis. The Vdr is highly expressed in pancreatic beta cells but also in the small and large intestine, in kidney tubular cells, in bronchial and skin epithelial cells and in osteoblast and osteocyte cells and in several types of immune cells, endocrine glands and certain reproductive tissues (Wang et al., 2012). Interestingly the expression of the Vdr in the liver, brain tissue and muscle is very low or even not detectable (Wang et al., 2012).

It is assumed that vitamin D can influence the expression of genes that are linked to diverse biological processes, in particular those relevant for the cardiovascular system (Nagpal et al., 2005).

Investigations regarding mechanisms or the causal role of vitamin D in blood pressure regulation come from *in vitro* and animal studies. The renin-angiotensin-aldosterone-system (RAAS) is a key regulator of blood pressure (Herichova and Szantoova, 2013). Data from *in vitro* studies showed that the Vdr-calcitriol (1,25-dihydroxyvitamin D) complex can bind to the promotor of the renin gene and inhibit renin expression (Yuan et al., 2007). This was corroborated by data from cohort and cross-sectional studies that indicate an inverse association between plasma renin activity and vitamin D levels in normo- and hypertensive individuals (Tomaschitz et al., 2010; Vaidya et al., 2011). A suitable *in vivo* model to investigate the causal role of vitamin D for blood pressure regulation is mice lacking a functional Vdr gene. The Vdr is a nuclear receptor that mediates the cellular effects of vitamin D by binding to vitamin D response elements of target genes (DeLuca, 2004). Therefore, Vdr-lacking Vdr^–/–^ mice are an animal model that emulates vitamin D deficiency. Two studies reported an increased expression and activity of renin, hypertension, and cardiomyocyte hypertrophy in Vdr^–/–^ mice (Li et al., 2002; Xiang et al., 2005). Another study found that Vdr^–/–^ mice had a significant blood pressure reduction despite a 50% higher renin activity and cardiac hypertrophy (Simpson et al., 2007). These authors proposed a blood pressure-independent anti-hypertrophic activity of vitamin D in the heart. Despite significant effects on blood pressure, the consequences of these changes in cardiac function have not been investigated. Furthermore, the influence of high dietary calcium intake, which is necessary to normalize serum minerals in Vdr^–/–^ mice, on blood pressure remains unclear. Thus, we aimed to elucidate the causal role of Vdr on renin expression, blood pressure, and heart function via transthoracic echocardiography in a long-term study using defined diets. In contrast to previous studies, we included two groups of Vdr^+/+^ in the study to differentiate between vitamin D and mineral effects. We hypothesized that Vdr deficiency would be associated with hypertension and deteriorated heart function in mice.

## Materials and Methods

### Mice and Diets

The study followed the established guidelines for the care and handling of laboratory animals and was approved by the local council of Saxony-Anhalt (Landesverwaltungsamt, Halle [Saale], Germany, approval number: 42502-2-1313 MLU). All mice were housed pairwise in Makrolon cages in a room with controlled temperature (22 ± 2°C), humidity (50–60%), and artificial lighting (6 am–6 pm) and had free access to food and water. The lamps used in the animal facility were assessed for emission of ultraviolet B (UVB) light (Solarmeter, Genside, PA, United States); no measurable UVB irradiation was found in close proximity to the light source.

**FIGURE 1:**
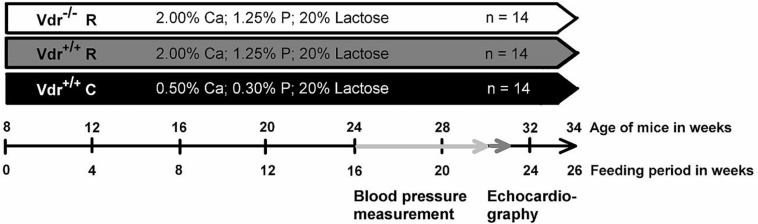
Diagram concerning the study procedure. The feeding period started when vitamin D receptor knockout (Vdr^–/–^) and Vdr^+/+^ mice were 8 weeks old. Mice were fed either a high-calcium (Ca), high-phosphorus (P) rescue diet (R) or a control diet (C) for 26 weeks. The training period of the blood pressure measurement for each mouse lasted 4 weeks and started at week 16 of the experiment. Subsequent to the training period, the blood pressure and heart rate were measured. The measurement period lasted 2 weeks. After 22 weeks of the study, the echocardiography was performed. At the end of the feeding period, at week 26, organs and blood were collected.

Homozygous Vdr^–/–^ mice and corresponding Vdr^+/+^ mice were obtained by mating heterozygous Vdr^ + ⁣/−^B6.129S4-Vdr^*tm1Mbd*^/J mice (Boston strain, Jackson Laboratories, Bar Harbor, ME, United States). Sixteen breeding pairs were used to obtain the required number of mice. All breeders received a commercial high-calcium (2%), high-phosphorus (1.25%) diet termed rescue diet (R) to prevent hypocalcemia, and a rickets phenotype secondary to Vdr deficiency. The commercial rescue diet contained 1,300 IU vitamin D_3_/kg and was fed before pregnancy, during pregnancy and also during lactation (Ssniff, Soest, Germany). Irrespective of the genotype, all offspring received the rescue diet until the age of 8 weeks. At the age of 8 weeks, male mice were subdivided into Vdr^–/–^ mice and Vdr^+/+^ mice. Fourteen Vdr^–/–^ mice (group 1) received a self-prepared high-calcium (2%), high-phosphorus (1.25%) rescue diet. Groups 2 and 3 that included 14 Vdr^+/+^ mice each, were fed either the rescue diet or a self-prepared control diet (C) containing 0.5% calcium and 0.3% phosphorus according to the recommendations of the American Institute of Nutrition (AIN) (Reeves et al., 1993). The two Vdr^+/+^ mice groups (Figure 1) were included to distinguish between the effects caused by calcium and phosphorus and those caused by vitamin D. Basal components of all purified diets were (in g/kg) corn starch (273), casein (200), sucrose (100), lactose (200), soybean oil (70), cellulose (50), DL-methionine (2), vitamin and mineral mixture (105). Lactose was added to both diets to improve intestinal calcium uptake (Li et al., 1998). Vitamins and minerals were added to the diets according to the recommendations of AIN (Reeves et al., 1993). All experimental diets contained 1,000 IU vitamin D_3_/kg diet. The three groups of mice were fed the experimental diets for 26 weeks. Body weights and feed intake were recorded weekly.

### Measurement of Blood Pressure and Heart Rate

Blood pressure was assessed non-invasively in all mice via external tail pulse detection (ADInstruments, Spechbach, Germany) in non-anesthetized mice. Prior to the actual measurement period, mice were trained to accustom them to this procedure. The training period lasted 4 weeks and started in the 16^th^ week of the study. The measurement period started in the 20^th^ week of the trial and lasted 2 weeks. The schedule of all measurements is shown in Figure 1. Assessment of blood pressure and heart rate occurred between 8 am and 12 am to reduce the influence of circadian rhythm.

### Echocardiography

Transthoracic echocardiography is an established non-invasive procedure, which is commonly used to assess cardiac morphology and performance (Gardin et al., 1995; Tanaka et al., 1996). Therefore, 30-week-old mice were anesthetized with 2% isoflurane (1 L/min O_2_) and transthoracic echocardiography was conducted by using a high-resolution imaging system for small animals (Vevo 2100, VisualSonics Inc., Canada) equipped with a high-frequency ultrasound probe (MS 550D). The heart was first imaged in the parasternal long axis in the two-dimensional mode. This view was used to position the M-mode cursor perpendicular to the ventricular septum and left ventricle (LV) posterior wall, after which the M-mode images were obtained. LV dimensions and wall thicknesses were determined from parasternal long axis M-mode images. LV ejection fraction (EF), LV fractional shortening (FS), ascending aortic peak velocity, and descending aortic peak velocity were calculated using Vevo 2100 software. Flow velocity and gradient in the aortic valve and descending aorta were determined from the aortic arch view in the pulsed wave Doppler mode. All parameters were determined from at least five heart cycles per mouse. A timeline is given in Figure 1.

### Sample Collection

At the age of 34 weeks, mice were food deprived for 4 h, anesthetized with diethyl ether, and sacrificed. Blood was collected into microtubes (Serum Z, Sarstedt, Nümbrecht, Germany) to obtain serum. Lungs and hearts with the adjacent aorta were prepared, rinsed with 0.9% NaCl solution, weighed, and snap-frozen in liquid nitrogen. The apex of the heart was fixed in 10% paraformaldehyde neutral buffered formalin solution (Sigma-Aldrich, St. Louis, MO, United States). Tibia length was assessed for normalization of organ weights.

### Analysis of Serum Parameters

Serum concentrations of calcium and inorganic phosphate were measured spectrophotometrically (Fluitest^®^ CA and Fluitest^®^ PHOS, Analyticon Biotechnologies AG, Lichtenfels, Germany). Serum iPTH and iFGF23 were quantified using ELISA kits (PTH: #60–2305, iFGF23: #60–6800; Immunotopics, San Clemente, CA, United States). Serum concentration of renin was analyzed by use of an ELISA Kit (ab193728, Abcam, Cambridge, United Kingdom). The renin activity was quantified by use of an angiotensin I ELISA kit (DB52011, IBL, Hamburg Germany). All analyses were performed according to the manufacturer’s specifications.

### Histological Analysis of the Heart

After fixation and several washing steps with PBS, apex of the heart was dehydrated using graded solutions of ethanol (30 min each in 10, 30, and 50% ethanol; overnight in 70% ethanol; and 30 min each in 90 and 100% ethanol). Samples were then infiltrated stepwise with PEG 1500 (Merck-Schuchhardt, Hohenbrunn, Germany) at 48°C and embedded in PEG 1500. Sections with 2 μm layer thickness were prepared with a standard rotary microtome (Microm HM 335 E, Thermo Fisher Scientific, Waltham, MA, United States) and mounted on poly-L-lysine-coated slides. Following staining with hematoxylin (Thermo Fisher Scientific), the sections were analyzed for myofibril area (Axiovert 200 microscope, Axiovision Rel. 4.8.2 software; Carl Zeiss, Jena, Germany). All samples were blinded during the analyses.

### RNA Isolation and Real-Time RT-PCR

Total RNA was extracted from the kidney to analyze the mRNA abundance of renin using Trifast reagent (VWR International, Radnor, PA, United States) according to the manufacturer’s protocol. Total RNA concentrations and purities were estimated by measuring the optical density at 260 and 280 nm, respectively. A total of 1.2 μg of total RNA were used for cDNA synthesis with M-MLV reverse transcriptase (Promega, Madison, WI, United States). Real-time RT-PCR analyses were performed as described elsewhere in detail (Schutkowski et al., 2014). For the determination of mRNA concentration, a threshold cycle (Ct) and amplification efficiency were obtained from each amplification curve using the Rotor-Gene software version 4.6 (Corbett Research, Mortlake, Australia). The relative mRNA concentration of renin was calculated according to Pfaffl (2001). Several reference genes were analyzed and their stable expression levels in all groups were evaluated by calculating Ct values. The most stable reference genes were used for normalization. These reference genes hypoxanthine guanine phosphoribosyl transferase (Hprt), ribosomal protein, large, P0 (Rplp0) and succinate dehydrogenase complex flavoprotein subunit A (Sdha). Characteristics of the primers are shown in Table 1.

**TABLE 1:** Primer characteristics.

**Genes**	**Primer sequences**
Angiotensin II receptor, type 1 (Agtr1)	forward 5′ – CGCTTCGGCCAGCGTCAGTT – 3′ reverse 5′ – GCCAAGCCAGCCATCAGCCA – 3′
Angiotensinogen (Agt)	forward 5′ – TGGGCTTCCGCATGTACAAGAT – 3′ reverse 5′ – GCAGTCTCCCTCCTTCACAG – 3′
Hypoxanthine guanine phosphoribosyl transferase (Hprt)	forward 5′ – AGGGATTTGAATCACGTTTG – 3′ reverse 5′ – TTTACTGGCAACATCAACAG – 3′
Mineralocorticoid receptor (Nr3c2)	forward 5′ – TTGGTGTGAATTCAGGTGGA – 3′ reverse 5′ – GTGACACCCAGAAGCCTCAT – 3′
Renin receptor (Atp6ap2)	forward 5′ – AGCTCCGTAACCGCCTGTTT – 3′ reverse 5′ – TCTACCACTGCGTTCCCACC – 3′
Ribosomal protein, large, P0 (Rplp0)	forward 5′ – GAAACTGCTGCCTCACATCCG – 3′ reverse 5′ – CTGGCACAGTGACCTCACACG – 3′
Succinate dehydrogenase complex flavoprotein subunit A (Sdha)	forward 5′ – GAATTTGTTCAGTTCCACCC – 3′ reverse 5′ – ATCTCAAGAGTCATGGATCG – 3′

### Western Blot

For western blotting tissue samples were lysed using RIPA buffer (50 mM Tris/HCl (pH 7.5), 150 mM NaCl, 1% Triton-X100, 0.5% sodium deoxycholate, 0.1% SDS, 5 mM EDTA containing a protease inhibitor mix) and tissue lyser. Protein concentration was determined according to Bradford. Protein lysates were separated on a SDS-PAGE. Afterward, proteins were transferred to nitrocellulose by semi-dry blotting. Glyceraldehyde-3-phosphate dehydrogenase (Gapdh) was used for normalization of protein expression data because its expression was not affected by the treatment. The following antibodies were used: anti-Agt1r (ABIN 6256893, Antikörper-Online, Aachen, Germany), anti p44/p42 Mapk (#4695, Cell Signaling Technology (CST), Danvers, MA, United States), anti-phosphorylated p42/44 Mapk (#4370, CST), anti-Gapdh (#5174, CST). Primary antibodies were detected using HRP-conjugated secondary antibodies [anti-rabbit IgG, (#7074, CST) or anti-mouse (#7076, CST)] using ECL Prime western blotting detection reagent (GE Healthcare, Munich, Germany).

### Statistics

Data were tested for normal distribution by the Shapiro–Wilk test and for homoscedasticity by the Levene’s test. Differences between the three groups were analyzed by one-way analysis of variance (ANOVA) if the data were normally distributed and the variances were homogenous. If ANOVA revealed significant differences, data were analyzed using Tukey’s *post hoc* tests. In case of variance heterogeneity, means of the three groups were analyzed using Welch’s ANOVA with the *post hoc* Games–Howell test. Means were considered significantly different at *P* < 0.05.

## Results

### Growth Performance and Markers of Mineral Status

**FIGURE 2:**
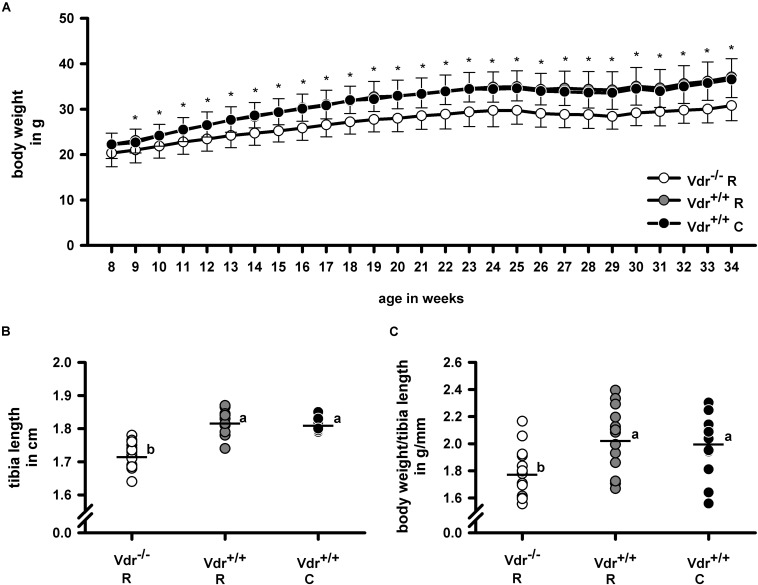
**(A)** Weight gain, **(B)** tibia length, and **(C)** body weight/tibia length-ratio of vitamin D receptor knockout (Vdr^–/–^) and Vdr^+/+^ mice. Body weight was measured weekly throughout the experimental period and tibia length was measured in 34-week-old Vdr^–/–^ and wild type (Vdr^+/+^) mice. Mice were fed either a high-calcium, high-phosphorus rescue diet (R) or a control diet (C) for 26 weeks. Values are means (*n* = 14). For body weight gain means and standard deviations (SD) are displayed. For tibia length and body weight/tibia length-ratio values are shown as individual data (∘) and means (−). Data were analyzed by one-way analysis of variance (ANOVA). For weight gain asterisks (^∗^) indicate significant differences between the Vdr^–/–^ R group and both Vdr^+/+^ groups. For tibia length and body weight/tibia length ratio values which do not share a common letter are significantly different (*P* < 0.05).

**FIGURE 3:**
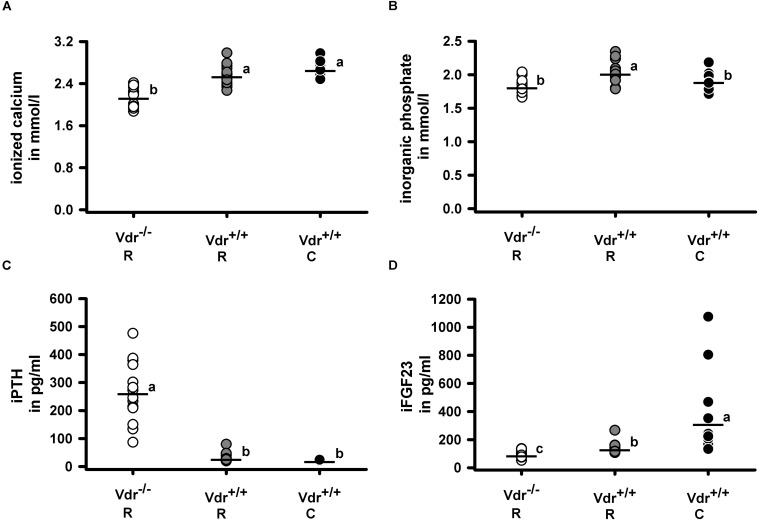
Serum concentrations of **(A)** ionized calcium, **(B)** inorganic phosphate, **(C)** intact parathyroid hormone (iPTH) and **(D)** intact fibroblast growth factor 23 (iFGF23). Serum metabolites and minerals were quantified in 34-week-old vitamin D receptor knockout (Vdr^–/–^) and Vdr^+/+^ mice. Mice were fed either a high-calcium, high-phosphorus rescue diet (R) or a control diet (C) for 26 weeks. Values are shown as individual data (∘) and means (−) (*n* = 14). Data were analyzed by one-way analysis of variance (ANOVA). Means not sharing a common letter are significantly different (*P* < 0.05).

Despite comparable energy intake (Vdr^–/–^ R: 52.7 ± 2.7 kJ/d; Vdr^+/+^ R: 51.7 ± 5.1 kJ/d; Vdr^+/+^ C: 51.4 ± 5.8 kJ/d), the final body weights (Figure 2A) of Vdr^–/–^ R mice were lower than those of mice from the Vdr^+/+^ groups. Furthermore, tibia length and the body weight/tibia length-ratio were smaller in the Vdr^–/–^ R mice than in both groups of Vdr^+/+^ mice (Figures 2B,C). To characterize the mineral status of these mice, serum concentrations of calcium and inorganic phosphate and hormones involved in regulation of calcium and phosphate homeostasis were analyzed. Data show that Vdr^–/–^ R mice had lower serum concentrations of calcium and higher concentrations of iPTH than both groups of Vdr^+/+^ mice (Figures 3A–C), indicating that the rescue diet was not capable of normalizing calcium status in Vdr^–/–^ R mice. The serum iPTH concentration in Vdr^–/–^ R mice showed marked interindividual variations ranging from normal to extremely high values (Figure 3C). Serum concentration of inorganic phosphate was higher in Vdr^+/+^ R mice than in mice from the Vdr^–/–^ R and Vdr^+/+^ C groups (Figure 3B). The phosphaturic hormone iFGF23 showed the lowest serum concentrations in Vdr^–/–^ R mice, followed by Vdr^+/+^ R mice and were highest in Vdr^+/+^ C mice (Figure 3D).

### Blood Pressure-Regulating Factors and Blood Pressure

**FIGURE 4:**
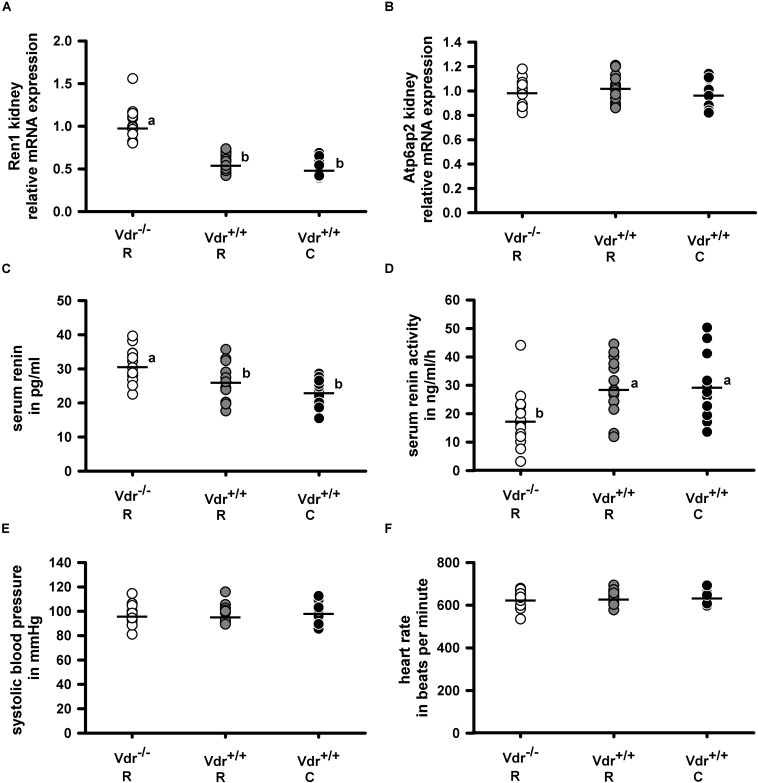
Relative mRNA expression of **(A)** renin (Ren1) and **(B)** renin receptor (Atp6ap2) in the kidney and **(C)** serum concentration of renin, **(D)** serum renin activity as well as **(E)** the systolic blood pressure and **(F)** heart rate in vitamin D receptor knockout (Vdr^–/–^) and Vdr^+/+^ mice. Mice were fed either a high-calcium, high-phosphorus rescue diet (R) or a control diet (C) for 26 weeks. Relative mRNA expression of Ren1 and Atp6ap2 in the kidney as well as the serum renin concentration and activity were measured at the end of the feeding period at the age of 34 weeks. Blood pressure and heart rate were measured at the age of 28–30 weeks. Values are shown as individual data (∘) and means (−) (*n* = 14). Data were analyzed by one-way analysis of variance (ANOVA). Means not sharing a common letter are significantly different (*P* < 0.05).

As Vdr is a transcription factor that influences gene and protein expression, we analyzed the mRNA expression of renin (Ren1) in the kidney and of angiotensinogen (Agt) in the liver as well as the circulating renin concentration in the three groups of mice. Figures 4A,C,D demonstrate that Vdr^–/–^ R mice had higher renal renin mRNA and higher serum renin protein concentrations but lower renin activity than both groups of Vdr^+/+^ mice. As proteolytic activity of renin increases after binding to the renin receptor (Atp6ap2), we analyzed the mRNA abundance of Atp6ap2 in kidney but found no differences between the groups (Figure 4B). Also the mRNA abundance of Agt in liver showed comparable values between the three groups of mice (Table 2). Despite differences in serum renin concentrations and serum renin activity, mice of the three groups did not differ in systolic blood pressure and heart rate (Figures 4E,F).

**TABLE 2:** Relative mRNA and protein expression of receptors involved in angiotensin II signaling in 34-week-old vitamin D receptor knockout (Vdr^–/–^) and Vdr^+/+^ mice.

**Parameters**	**Vdr^–/–^ R**	**Vdr^+/+^ R**	**Vdr^+/+^ C**	**P**
***Heart (n = 7)***				
*Relative protein expression (housekeeping protein: Gapdh)*				
Agt1r	1.00 ± 0.21	0.93 ± 0.22	1.04 ± 0.20	ns
p42 Mapk	1.00 ± 0.17	1.00 ± 0.15	0.97 ± 0.11	ns
p-p42 Mapk/p42 Mapk	1.00 ± 0.40	1.67 ± 1.48	1.05 ± 0.75	ns
p44 Mapk	1.00 ± 0.20	0.93 ± 0.11	0.95 ± 0.11	ns
p-p44 Mapk/p44 Mapk	1.00 ± 0.27	1.41 ± 0.71	0.99 ± 0.33	ns
***Kidney (n = 14)***				
*Relative mRNA expression (housekeeping genes: Rplpo0/Hprt)*				
Nr3c2	1.00 ± 0.15	1.03 ± 0.19	1.11 ± 0.17	ns
*Relative protein expression (housekeeping protein: Gapdh)*				
Agt1r	1.00 ± 0.43	0.77 ± 0.38	0.72 ± 0.32	ns
p42 Mapk	1.00 ± 0.38	0.95 ± 0.27	1.09 ± 0.37	ns
p-p42 Mapk/p42 Mapk	1.00 ± 0.49	0.98 ± 0.77	0.87 ± 0.41	ns
p44 Mapk	1.00 ± 0.37	0.99 ± 0.28	1.12 ± 0.41	ns
p-p44 Mapk/p44 Mapk	1.00 ± 0.52	0.91 ± 0.71	0.91 ± 0.51	ns
***Liver (n = 14)***				
*Relative mRNA expression (housekeeping genes: Sdha/Hprt)*				
Agt1r	1.00 ± 0.20	0.91 ± 0.09	0.90 ± 0.17	ns
Nr3c2	1.00 ± 0.19	0.91 ± 0.19	0.88 ± 0.17	ns
Agt	1.00 ± 0.19	1.03 ± 0.16	1.00 ± 0.17	ns
*Relative protein expression (housekeeping protein: Gapdh)*				
p42 Mapk	1.00 ± 0.16	0.94 ± 0.17	0.97 ± 0.18	ns
p-p42 Mapk/p42 Mapk	1.00 ± 0.57	0.79 ± 0.43	0.70 ± 0.37	ns
p44 Mapk	1.00 ± 0.19	1.01 ± 0.21	1.02 ± 0.25	ns
p-p44 Mapk/p44 Mapk	1.00 ± 0.68	0.89 ± 0.48	0.87 ± 0.50	ns

*Data are presented as means ± standard deviation (*n* = 14). Mice were fed either a high-calcium, high-phosphorus rescue diet (R) or a control diet (C) for 26 weeks. Data were analyzed by one-way analysis of variance (ANOVA). ns, not significant (*P* > 0.05). Agt, angiotensinogen; Agtr1, angiotensin II receptor, type 1; Gapdh, glyceraldehyde-3-phosphate dehydrogenase; Hprt, hypoxanthine guanine phosphoribosyl transferase; Mapk, Map kinase; Nr3c2, mineralocorticoid receptor, p-p42/p44 Mapk, phosphorylated p42/p44 Mapk; Rplp0, ribosomal protein, large, P0; Sdha, succinate dehydrogenase complex flavoprotein subunit A.*

To find mechanisms that may explain the normal blood pressure in the Vdr^–/–^ mice despite their higher renin concentrations in serum, we measured the expression of several other blood pressure-regulating factors such as the angiotensin II receptor (Agt1r) and the active p42/44 Mapk in heart, kidney and liver, as well as the renal and hepatic mineralocorticoid receptor (Nr3c2) but found no differences between the three groups of mice (Table 2).

### Morphometric and Functional Heart Parameters

To elucidate vitamin D effects on hearts independently of blood pressure values, we assessed cardiac myocyte size and heart function via transthoracic echocardiography. We found that heart weight relative to body weight was higher in Vdr^–/–^ R mice than in Vdr^+/+^ mice of both groups. However, these differences disappeared when heart weight was based on tibia length (Table 3). The lung weight also did not differ between the three groups of mice. To elucidate the role of Vdr in heart morphology and function, cardiac myocyte size and heart function were analyzed. Histological analysis did not reveal differences in cardiac myocyte size and LV thickness of mice from the three groups (Table 4) and thoracic echocardiography measurements also did not indicate a Vdr effect on systolic heart function, because the left ventricular ejection fraction, fractional shortening, posterior wall thickening, and blood flow velocity were comparable between Vdr^–/–^ R and Vdr^+/+^ mice (Table 4).

**TABLE 3:** Ratio of heart weight (HW) and lung weight (LW) to body weight (BW) as well as HW and LW to tibia length (TL), myocyte area and weight of the lungs in 34-week-old vitamin D receptor knockout (Vdr^–/–^) and Vdr^+/+^ mice.

**Parameters**	**Vdr^–/–^R**	**Vdr^+/+^ R**	**Vdr^+/+^ C**	**P**
HW (mg) / BW (g)	4.43^a^ ± 0.36	3.83^b^ ± 0.42	3.70^b^ ± 0.43	<0.001
HW (mg) / TL (mm)	7.87 ± 0.30	7.76 ± 0.95	7.38 ± 0.54	ns
Myofibril area (μm^2^)	378 ± 119	327 ± 59	318 ± 27	ns
LW (mg)	143 ± 9	151 ± 12	143 ± 10	ns
LW (mg) / BW (g)	4.69 ± 0.55	4.08 ± 0.30	3.97 ± 0.61	ns
LW (mg) / TL (mm)	8.31 ± 0.59	8.27 ± 0.66	7.89 ± 0.54	ns

*Data are presented as means ± standard deviation (*n* = 14); Mice were fed either a high-calcium, high-phosphorus rescue diet (R) or a control diet (C) for 26 weeks. Data were analyzed by one-way analysis of variance (ANOVA). Means not sharing a common letter are significantly different (*P* < 0.05).*

**TABLE 4:** Echocardiographic measurements of the interventricular septal (IVS) wall thickness, left ventricle internal dimension (LVID), and the left ventricle posterior wall (LVPW) thickness measured at end diastole or end systole in 30-week-old vitamin D receptor knockout (Vdr^–/–^) and Vdr^+/+^ mice.

**Parameters**	**Vdr^–/–^R**	**Vdr^+/+^ R**	**Vdr^+/+^ C**	**P**
**In mm**				
IVS wall thickness at end diastole	0.99 ± 0.31	1.03 ± 0.20	1.01 ± 0.18	ns
IVS wall thickness at end systole	1.34 ± 0.29	1.40 ± 0.29	1.41 ± 0.23	ns
LVID at end diastole	3.49 ± 0.44	3.47 ± 0.48	3.40 ± 0.44	ns
LVID at end systole	2.29 ± 0.42	2.19 ± 0.52	2.14 ± 0.41	ns
LVPW thickness at end diastole	1.00 ± 0.38	1.11 ± 0.34	1.05 ± 0.39	ns
LVPW thickness at end systole	1.38 ± 0.36	1.49 ± 0.31	1.40 ± 0.36	ns
**In %**				
LV fractional shortening	34.6 ± 7.2	37.7 ± 7.7	37.4 ± 6.7	ns
LV ejection fraction	64.1 ± 9.3	68.1 ± 9.7	68.1 ± 8.2	ns
LV posterior wall thickening	45.7 ± 19.0	37.7 ± 15.1	41.0 ± 9.2	ns
**In mm/s**				
Ascending aorta peak velocity	1410 ± 331	1273 ± 308	1167 ± 398	ns
Descending aorta peak velocity	−730 ± 173	−771 ± 246	-689 ± 182	ns

*Data are presented as means ± standard deviation (*n* = 14); Mice were fed either a high-calcium, high-phosphorus rescue diet (R) or a control diet (C) for 26 weeks. Data were analyzed by one-way analysis of variance (ANOVA). ns, not significant (*P* > 0.05). Measurements were made in M-Mode from the parasternal long axis.*

## Discussion

The causal role of vitamin D for regulating blood pressure and cardiac function is still elusive. To gain more insight into the role of vitamin D in cardiovascular health, we conducted a long-term study using Vdr^–/–^ mice as an animal model of Vdr deficiency and defined diets.

The finding that Vdr^–/–^ mice have higher renin mRNA expression in the kidney confirms data that propose that the Vdr is a negative regulator of renin mRNA expression (Li et al., 2002). Furthermore, Vdr^–/–^ mice from this study were found to have significantly higher concentrations of circulating renin than the Vdr^+/+^ mice, which validate the data obtained from previous studies (Li et al., 2002; Xiang et al., 2005; Simpson et al., 2007). However, it was surprising that Vdr^–/–^ mice compared to Vdr^+/+^ mice had a lower renin activity which reflect the capacity of renin to generate angiotensin I. Renin activity is affected by several factors, including blood pressure, sympathetic nervous system and sodium balance (Brown, 2007). Recent data show that 1α(OH)ase knockout mice deficient in biological active vitamin D develop diuresis and polydipsia because of a disturbed expression of aquaporins (Fu et al., 2019). Thus, it can be assumed that vitamin D deficiency may change water and sodium balance.

However, the higher serum concentration of renin and the lower renin activity were not associated with an altered blood pressure in these mice. This result is in contrast to previous data that showed increased blood pressure in transgenic mouse models lacking the Vdr or calcitriol synthesizing 1α(OH)ase (Li et al., 2002; Zhou et al., 2008) or that observed a reduction of blood pressure in hypertensive rats supplemented with vitamin D (Borges et al., 1999). In contrast, Simpson et al. observed a lower blood pressure in 9-month-old Vdr^–/–^ mice in comparison to Vdr^+/+^ mice (Simpson et al., 2007). Another study conducted with rats reported a U-shaped relationship between blood pressure and serum levels of vitamin D, with high blood pressure levels at very low and very high circulating levels of vitamin D (Mirhosseini et al., 2016). It should be noted that Li et al. who found an increase of blood pressure in Vdr^–/–^ mice used a normal chow diet for the first 8 weeks *post partum* before switching to a rescue diet (Li et al., 2002). In contrast, Simpson et al. who observed a reduction of blood pressure in 9-month-old Vdr^–/–^ mice administered the rescue diet throughout the complete life of mice (Simpson et al., 2007). We assume that differences in the experimental conditions, in particular the administered type of diet, can influence the blood pressure of mice and could partly explain the different results obtained from those studies.

Blood pressure is regulated by numerous factors such as cardiac output, blood volume, neurohumoral mechanisms and vascular resistance. The change in one regulating factor such as renin or its activity does not necessarily mean a change in blood pressure. To elucidate whether vitamin D can influence other blood pressure regulating pathways, we analyzed angiotensin II receptor type 1, angiotensinogen, mineralocorticoid receptor, renin receptor and p42/44 Map kinase expression but found no differences in the expression of receptors that are involved in angiotensin II and aldosterone signaling and the control of sodium homeostasis. The current finding that the Vdr does not play a crucial role in modification of the blood pressure is confirmed by recent meta-analysis of human randomized controlled intervention studies that found no changes of blood pressure and vascular function in individuals who received vitamin D (Beveridge et al., 2015, 2018; Wu and Sun, 2017).

To evaluate the resting blood pressure of the mice several precautions were taken. To exclude negative impacts of narcosis, we performed tail-cuff measurements in awake mice that were thoroughly trained for this procedure. Thereby, we avoided blood pressure increases due to stress. Additionally, we performed the measurements during the sleeping period of the mice (light period 6 am–6 pm), because during this period, the blood pressure of mice is at the diurnal minimum; this explains that the systolic blood pressure in these animals was about 100 mmHg.

Calcium, which is assumed to impact blood pressure, could have been another modifying factor (Kunutsor and Laukkanen, 2017). We observed that Vdr^–/–^ mice had lower serum levels of ionized calcium than Vdr^+/+^ mice, despite feeding them a high-calcium rescue diet. This is in contrast to a previous study that found normal concentrations of serum minerals in Vdr^–/–^ mice that received a normal rodent chow diet (Xiang et al., 2005). The difference in serum calcium, which had already been observed in a recent study using Vdr^–/–^ mice (Grundmann et al., 2017) is an additional factor that could have influenced blood pressure. The finding that serum concentrations of the calcium-sensor iPTH and the phosphate-sensor FGF23, which are central regulators of serum mineral homeostasis, were significantly different between both genotypes further supports that the serum mineral levels were not normalized by feeding a rescue diet in the current study.

Several mechanisms of calcium on blood pressure have been postulated: (i) calcium can increase the contractility of vascular smooth muscle cells and in turn vasoconstriction, (ii) calcium can mediate the release of catecholamines, (iii) low extracellular calcium levels stimulate renin secretion thereby increasing angiotensin II formation, and (iv) calcium can impact areas of blood pressure regulation in the central nervous system (Beierwaltes, 2010; Kunutsor and Laukkanen, 2017). Thus, it can be assumed that low serum calcium concentration in Vdr^–/–^ mice could have interfered with renin synthesis or action. This would explain the contradictory findings regarding blood pressure of Vdr^–/–^ mice, because some mice in the group that was fed the rescue diet did not develop hypocalcemia (Li et al., 2002; Simpson et al., 2007).

In addition to a possible influence of calcium on blood pressure, studies in rats show an influence of maternal vitamin D supply. It is described that vitamin D deficiency in the womb resulted in hypertension, endothelial dysfunction and impaired smooth muscle cell function of the offspring (Tare et al., 2011). In addition, limited maternal vitamin D supply during the renal development phase leads to increased renal renin gene expression and also affects the renal function in males (Boyce et al., 2013). This raises the importance of maternal vitamin D supply. Possible differences between the blood pressure outcomes in studies listed above could therefore also be attributed to a different maternal vitamin D supply. Since all breeders received a diet with the recommended amount of 1,300 IU vitamin D/kg diet in the current study, maternal vitamin D deficiency is not to be expected.

To our knowledge, Vdr^–/–^ mice do not have any disturbed renal function. Vitamin D deficiency during the development period of the nephron simulates nephrogenesis (Maka et al., 2008; Nascimento et al., 2012). Kong et al. who have conducted a study with Vdr^–/–^ mice showed that these mice are not suffering from an impaired renal fluid handling, but had developed polyuria (Kong et al., 2008). Thus, the polyuria could have contributed to a lower blood volume and the normalization of blood pressure in the Vdr^–/–^ mice used in our study. However, regulation of blood pressure is a complex network. Besides the RAAS system, catecholamines, nitric oxide, prostaglandins, baroreceptors and several other factor can influence blood pressure.

To elucidate whether Vdr deficiency was associated with cardiac hypertrophy or impaired cardiac function independently of blood pressure, we assessed heart weight, cardiac myocyte morphology, and heart function via echocardiography. Findings from a previous study that revealed that Vdr^–/–^ mice have an increased energy expenditure due to alopecia were confirmed (Schutkowski et al., 2018). As we did not find any differences in blood pressure, cardiac morphology and heart function between Vdr^–/–^ and Vdr^+/+^ mice, we assume that vitamin D does not have an independent causal role in cardiac morphology. This is in contrast with data from previous studies that were able to find anti-hypertrophic effects of vitamin D (Wu et al., 1996; Bodyak et al., 2007; Choi et al., 2011), but would explain why vitamin D is not associated with an improvement of cardiovascular health in most human intervention trials.

The strengths of our study were: (i) the long study period (26 weeks); (ii) the inclusion of two groups of Vdr^+/+^ mice that received a normal and high-calcium, high-phosphorus diet to distinguish between vitamin D and mineral effects; (iii) the use of defined and clearly characterized basal diets; (iv) the long training period prior to the actual measurement of blood pressure; and (v) the assessment of heart function. However, our study has a weakness in that we analyzed the role of Vdr deficiency and not vitamin D deficiency through dietary depletion. Originally, vitamin D was thought to act by altering the expression of target genes via the Vdr. Meanwhile, vitamin D has also been shown to have receptor-independent effects (Norman et al., 1992). Thus, our study design does not allow us to exclude that vitamin D might have Vdr-independent effects.

To conclude, the current findings did not indicate the role of a functional Vdr on the regulation of blood pressure and heart function under these experimental conditions. Whether low calcium serum levels, which we observed in Vdr^–/–^ mice, can counteract the effect of increased circulating renin on blood pressure remains to be elucidated.

## Data Availability

The raw data supporting the conclusions of this manuscript will be made available by the authors, without undue reservation, to any qualified researcher.

## Ethics Statement

This study was carried out in accordance with the recommendations of the established guidelines for the care and handling of laboratory animals and was approved by the local council of Saxony-Anhalt [Landesverwaltungsamt, Halle (Saale), Germany, approval number: 42502-2-1313 MLU].

## Author Contributions

SG, BK, and GS conceived and designed the study. SG performed the experimental study. MG and BS supervised the blood pressure measurement and the echocardiography. SG, BS, BK, and SR performed the blood pressure measurement and the echocardiography. SG performed the serum analyses and analyzed the data. AS performed the histological analysis. SG, AS, and GS wrote the manuscript.

## Conflict of Interest Statement

The authors declare that the research was conducted in the absence of any commercial or financial relationships that could be construed as a potential conflict of interest.

## Abbreviations

25(OH)D: 25-hydroxyvitamin D
AIN: American Institute of Nutrition
ANOVA: one-way analysis of variance
C: control
CVD: cardiovascular diseases
EF: ejection fraction
FS: fractional shortening
GAPDH: glyceraldehyde-3-phosphate dehydrogenase
Hprt: hypoxanthine guanine phosphoribosyl transferase
iFGF23: intact fibroblast growth factor 23
iPTH: intact parathyroid hormone
LV: left ventricle
PBS: phosphate buffered saline
R: rescue
RAAS: renin-angiotensin-aldosterone-system
Rplp0: ribosomal proteinlarge,P0
Vdr: vitamin D receptor.
